# Antibody induced CD4 down-modulation of T cells is site-specifically mediated by CD64^+^ cells

**DOI:** 10.1038/srep18308

**Published:** 2015-12-16

**Authors:** Stephanie Vogel, Elena Grabski, Daniela Buschjäger, Frank Klawonn, Marius Döring, Junxi Wang, Erika Fletcher, Ingo Bechmann, Torsten Witte, Martin Durisin, Burkhart Schraven, Sara M. Mangsbo, Kurt Schönfeld, Niklas Czeloth, Ulrich Kalinke

**Affiliations:** 1 Institute for Experimental Infection Research, TWINCORE, Centre for Experimental and Clinical Infection Research, a joint venture between the Helmholtz Centre for Infection Research (HZI) and the Hannover Medical School (MHH), Feodor-Lynen-Straße 7, D30625 Hannover; 2 Department of Computer Science, Ostfalia University of Applied Sciences, Salzdahlumer Straße 46/48, D38302 Wolfenbüttel, Germany; 3 Biostatistics, Helmholtz Centre for Infection Research, Inhoffenstraße 7, D38124 Braunschweig, Germany; 4 Department of Immunology Genetics and Pathology, Uppsala University, Rudbeck Laboratory, S75185 Uppsala, Sweden; 5 Immuneed AB, S-756 52, Uppsala, Sweden; 6 Institute for Anatomy, University Leipzig, Liebigstraße 13, D04103 Leipzig; 7 Clinic for Immunology and Rheumatology, Hannover Medical School, Carl-Neuberg-Straße 1, D30625 Hannover, Germany; 8 Department of Otolaryngology, Hannover Medical School, Carl-Neuberg-Straße 1, D30625 Hannover, Germany; 9 Institute for Molecular and Clinical Immunology, Medical Faculty, Otto-von-Guericke University, D39120 Magdeburg, Germany; 10 Department of Immune Control, Helmholtz Centre for Infection Research, Inhoffenstrß2 7, D38124 Braunschweig, Germany; 11 Biotest AG, Dreieich, Landsteinerstrasse 5, D63303 Dreieich, Germany

## Abstract

Treatment of PBMC with the CD4-specific mAb BT-061 induces CD4 down-modulation of T cells. Here we report that addition of BT-061 to purified T cells did not confer this effect, whereas incubation of T cells in BT-061 coated wells restored CD4 down-modulation. These results implied that Fcγ receptor mediated cell-cell interactions played a role. In consistence with this hypothesis PBMC depleted of CD64^+^ monocytes did not confer CD4 down-modulation of BT-061 decorated T cells. Strikingly, CD4 down-modulation was observed in BT-061 treated synovial fluid punctuated from patients’ inflamed joints that comprised enhanced numbers of CD64^+^ cells. In contrast, in a circulating whole blood system injection of BT-061 did not induce CD4 down-modulation, due to CD64 saturation by serum IgG. Similarly, tonsil derived mononuclear cells devoid of CD64^+^ cells did not show CD4 down-modulation, whereas addition of blood derived monocytes restored the effect. Thus, the interaction of BT-061 decorated T cells with CD64^+^ cells is needed for CD4 down-modulation, implying that in patients BT-061 would primarily induce CD4 down-modulation at inflammatory sites. These results highlight the need not only to examine the interaction of a given mAb with single FcγR, but also the immunological environment that is appropriate to support such interactions.

Currently, a whole variety of different monoclonal antibodies (mAbs) is being developed for the therapy of diverse diseases, such as cancer and autoimmune conditions. mAb function mostly is conferred by (i) depletion of target cells, (ii) inhibition of cell-cell or cell-ligand interactions, or (iii) agonistically triggering signals that affect cell function, e.g. to suppress activity of self-reactive T cells. Protein engineering offers the possibility to tailor mAb formats for given therapeutic purposes^1^. However, to be able to fully exploit such technologies, a detailed understanding of the mAb effector function is required^2^. In the past, selection of the constant region (C region) for therapeutic mAbs was based on knowledge retrieved from *in vitro* analysis of effector functions such as phagocytosis, induction of inflammatory cytokines or chemokines, and antibody-dependent cellular cytoxicity (ADCC). These effector functions are dependent on the interaction with Fcγ receptors (FcγR) expressed by immune cells. The family of human FcγR comprises three major classes, FcγRI (CD64), FcγRII (CD32) and FcγRIII (CD16), each one showing distinct structural and functional properties^3^. Based on their affinity for monomeric IgG the receptors are divided into high affinity CD64 and medium to low affinity CD32 and CD16^3^,^4^. Upon engagement, the receptors signal via immunoreceptor tyrosine-based activating (ITAM) or inhibitory (ITIM) motifs that are comprised within the cytoplasmic tail of the receptors or that are associated with signaling adaptors^5^,^6^.

Recent data underscored that FcγR antibody interactions determined by standard *in vitro* methods, such as binding studies by Biacore techniques, may not fully capture *in vivo* relevant effects. One dramatic example is the incidence with the superagonistic mAb TGN1412. On the basis of methods that were available in those days this IgG4 mAb was predicted not to show significant interactions with FcγR. However, in 6 healthy individuals treatment with 0.1 mg/kg of this antibody induced a life threatening cytokine storm^7^. In the last years we and others found that TGN1412 binding of T cells alone did not induce massive cytokine release, whereas interaction via FcγR CD32B expressed e.g. by B cells dramatically boosted T cell activation^8^,^9^. On the one hand this experience highlighted the need for more detailed preclinical *in vitro* studies that truly reflect *in vivo* conditions and thus allow better prediction of mAb effects in humans. On the other hand not only target-specific mAb properties, but also Fc mediated effects, are important and must be well understood.

The interaction between mAb and FcγR is determined by the subclass of the antibody and by the glycosylation of the antibody, which is dependent on the system the mAb was produced in^10^. Because marginal modifications might massively influence the efficacy of a mAb, every new therapeutic mAb should thoroughly be examined *in vitro* concerning its FcγR interactions, focusing especially on the compartments this interaction will likely take place in. This is of particular importance because conditions in healthy individuals and patients may differ with respect to e.g. FcγR expression patterns or blood parameters such as IgG levels and abundance of single cell subsets, which can significantly influence mAb - FcγR interactions. The need of thorough analysis of mAb actions was additionally highlighted by recent publications in the field of cancer research as it was found that the tumor microenvironment as well as FcγR interactions have a strong impact on the activity of immunomodulatory antibodies^11^,^12^.

Therefore, here we analyzed the anti-CD4 mAb BT-061 (Tregalizumab), which currently is under development for the treatment of autoimmune diseases such as rheumatoid arthritis and psoriasis^13^. This mAb was generated by grafting the complementarity determining regions of the murine CD4-specific mAb hB-F5 into a human IgG1κ antibody^14^. In *in vitro* assays BT-061 activated regulatory T cells in a Lck dependent manner^15^ and conferred CD4 down-modulation^16^. However, which Fcγ receptor is targeted by this IgG1 monoclonal antibody and especially in which compartment Fcγ receptor interactions can take place was not known. Therefore, here we analyzed BT-061 induced CD4 down-modulation in PBMC, in synovial fluid, in synovial fluid derived cell preparations, in a circulating whole blood system, and in tonsil derived mononuclear cell preparations, as a read-out to characterize IgG1 mediated Fcγ receptor interactions. Our results indicate that in addition to CD4 binding interaction with CD64^+^ cells is needed to confer BT-061 mediated CD4 down-modulation of T cells. Strikingly we found that these interactions cannot be achieved in the blood stream or secondary lymphoid tissues, but only at sites of inflammation where abundant CD64 expressing cells are present.

## Results

### BT-061 treatment of PBMC induces CD4 down-modulation of T cells

**Figure 1:**
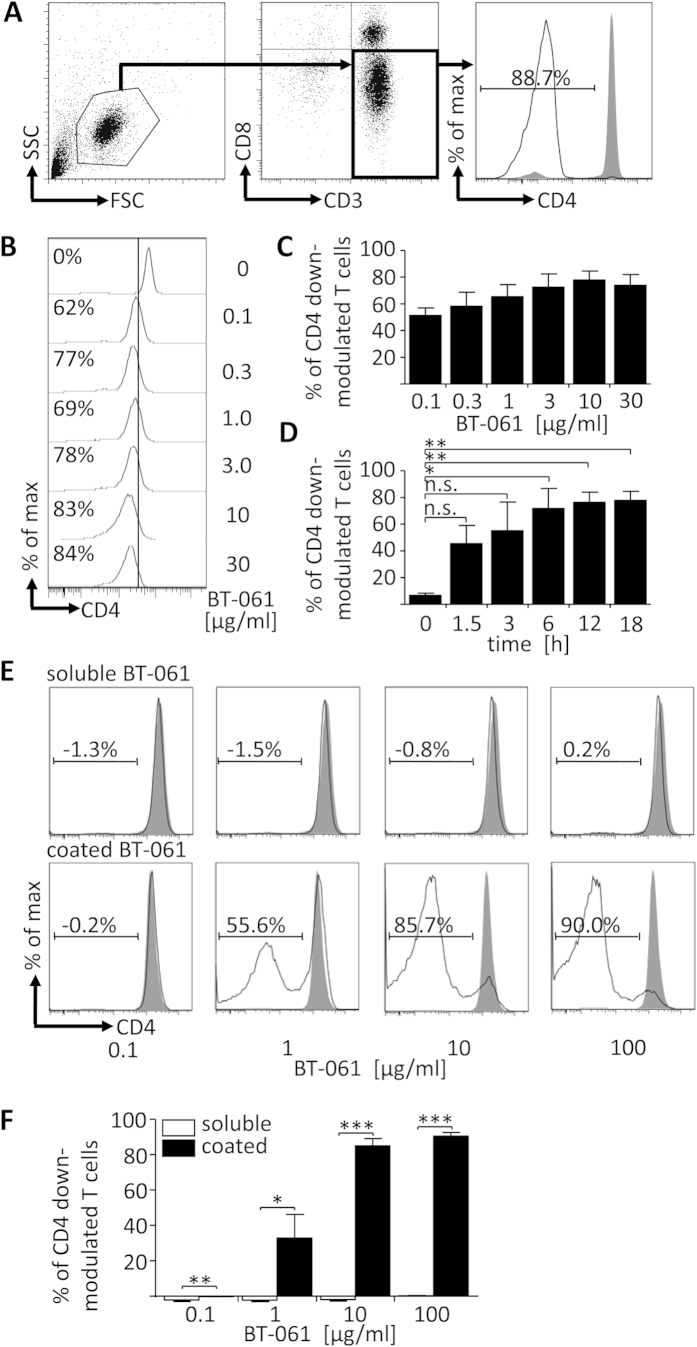
BT-061 treatment of PBMC induces CD4 down-modulation of T cells. (**A**) CD3^+^ T cells were stained with anti-CD3, anti-CD8a, and anti-CD4 clone SK3 that binds a different epitope than BT-061 (filled grey), or an isotype control (black line). In cytometric analysis CD3^+^CD8^-^ T cells were defined as CD4 down-modulated and their percentage was calculated. (**B**) PBMC were treated with the indicated concentrations of BT-061 for 45 min at 4 °C and then cultivated for 18 h at 37 °C. CD4 expression of T cells was determined cytofluometrically. One experiment of 2 similar ones with cells derived from 3–5 donors is shown. (**C**) Statistical analysis from all experiments as shown in (**B**). (**D**) PBMC were treated with BT-061 (10 μg/ml) for 45 min at 4 °C and then cultivated for the indicated times at 37 °C. CD4 expression was determined cytofluometrically. Statistical analysis of 2 similar experiments with cells derived from 3–5 donors. (**E**) Purified CD3^+^ T cells were treated with BT-061 (as in B, black line) or with medium (filled grey) and then incubated for 18 h at 37 °C (upper row). Purified CD3^+^ T cells were cultured for 18 h at 37 °C in wells coated with BT-061 at the indicated concentrations (black line) or in untreated wells (filled grey) (lower row). CD4 expression was determined cytofluometrically. Representative data of one experiment from cells derived from 3 donors (upper row) or from 2 similar experiments with cells derived from 5 donors (lower row) are shown. (**F**) Statistical analysis from all experiments as shown in (**E**). Error bars indicate SEM.

To analyze whether Fcγ receptor interactions of BT-061 bound to T cells played a role in conferring CD4 down-modulation, the CD4 expression of T cells was determined cytofluometrically on CD3^+^CD8^–^ lymphocytes using the CD4-specific mAb SK3, which recognizes a different epitope than BT-061 (Fig. 1A). Upon 18 h incubation of PBMC at 37 °C with different concentrations of soluble BT-061, T cells showed CD4 down-modulation that gradually increased with rising BT-061 concentrations and that reached a plateau at 10 μg/ml (Fig. 1B,C). Analysis of the kinetics of CD4 down-modulation revealed that already 1.5 h after incubation with 10 μg/ml BT-061 considerable CD4 down-modulation was detected that peaked after 18 h (Fig. 1D). Importantly, this loss of CD4 was not accounted by depletion or ADCC of T cells because in the CD4 down-modulation assay the absolute number of CD3^+^ cell stayed constant (Supplementary Fig. 1). Addition of soluble BT-061 to MACS enriched T cells did not induce CD4 down-modulation (Fig. 1E, upper panel, and F), whereas purified T cells cultivated in wells coated with BT-061 showed strong CD4 down-modulation (Fig. 1E, lower panel, and F). These results indicated that BT-061 binding of T cells alone was not sufficient to induce CD4 down-modulation and that BT-061 decorated T cells had to interact with some immune cell subset comprised within PBMC in order to show the effect.

Because previously it was shown that BT-061 decorated T cells treated with a cross-linking secondary mAb showed Lck phosphorylation^16^, we next addressed whether also in BT-061 treated PBMC CD4 down-modulation of T cells was dependent on agonistic signaling. To this end we used the Src-family tyrosine kinases inhibitor PP1, which inhibits T cell receptor (TCR) triggering-dependent phosphorylation of the lymphocyte protein-tyrosine kinase (Lck)^17^. PP1 pre-treatment of PBMC reduced BT-061 mediated CD4 down-modulation (Supplementary Fig. 2A and B) indicating that also in BT-061 treated PBMC CD4 down-modulation was at least partially dependent on Lck activation.

### Fcγ-receptor mediated cell-cell-contact with CD64^+^ monocytes confers efficient CD4 down-modulation of BT-061 decorated T cells

**Figure 2:**
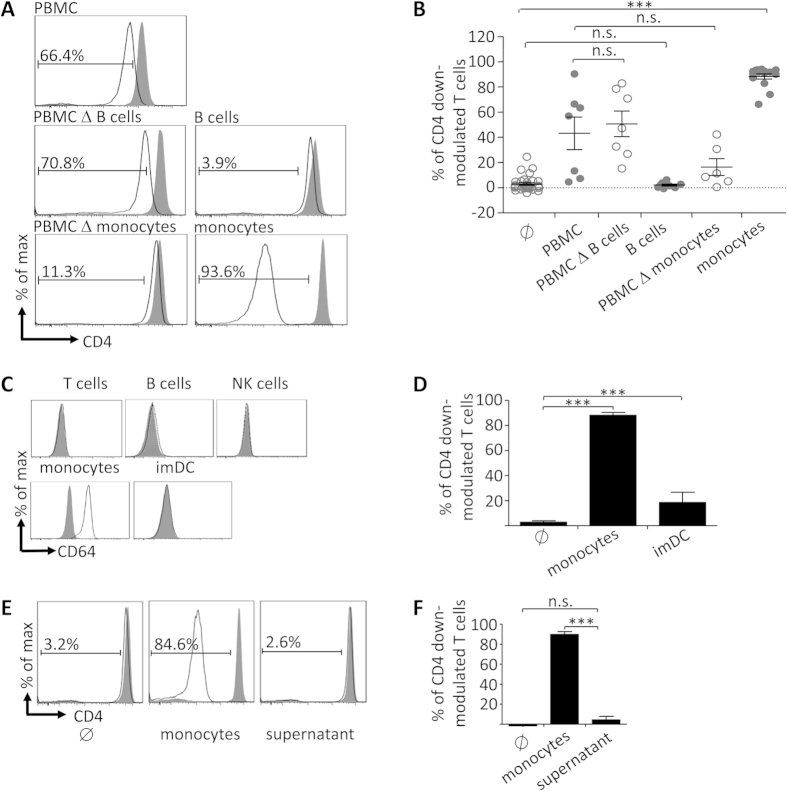
Monocytes confer robust CD4 down-modulation of BT-061-decorated T cells by cell-cell-contact. (**A**) BT-061 decorated (black line) or medium treated (filled grey) CD3^+^ T cells were cultivated for 18 h at 37 °C either in medium (∅), with PBMC, with immune cell subsets (as indicated), or with PBMC depleted of the respective immune cell subsets. Representative data are shown of 4 experiments performed with cells from 7 donors (PMBC), 4–5 experiments with cells from 7–8 donors (B cell depleted PBMC or B cells), and 4–8 experiments with cells from 6–15 donors (monocytes depleted PBMC or monocytes). (**B**) Statistical analysis from the experiments in (**A**). (**C**) CD3^+^ T cells, CD19^+^ B cells, CD56^+^ NK cells, CD14^+^ monocytes and HLA-DR^+^ CD14^–^ immature moDC (imDC) were stained for CD64 (black lines) or isotype control (filled grey). Representative data from 3 experiments are shown. (**D**) BT-061 decorated T cells were co-cultured with monocytes or imDC for 18 h at 37 °C. Statistical analysis of 4–8 experiments with cells from 6–15 (monocytes) and one experiment with cells from 4 donors (imDC). Error bars indicate SEM. (**E**) BT-061 decorated (black line) or medium treated (filled grey) CD3^+^ T cells were cultivated for 18 h at 37 °C together with monocytes (middle panel), or incubated in cell-free supernatant taken from BT-061 decorated T cells co-incubated with monocytes for 18 h (right panel). Data are shown of two experiments with cells from 3 donors. (**F**) Statistical analysis from all experiments as shown in (**E**).

**Figure 3:**
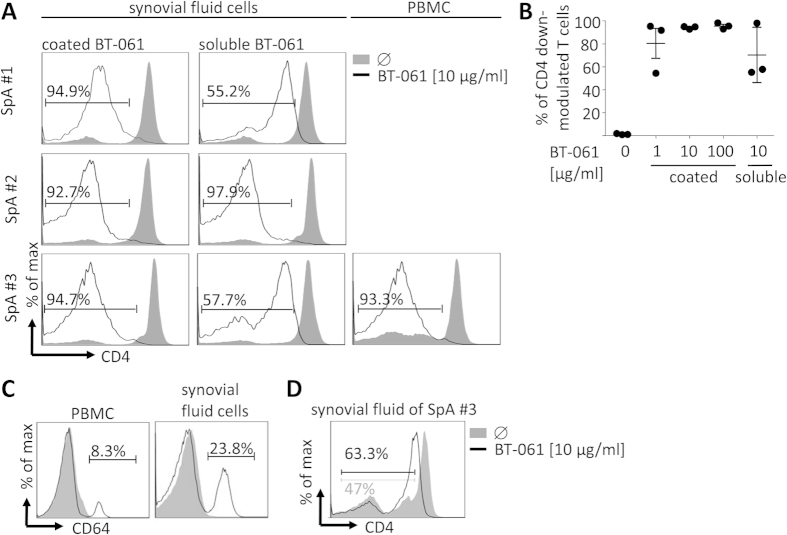
BT-061 treatment of synovial-fluid-derived mononuclear cells and whole synovial fluid induces CD4 down-modulation of T cells. (**A**) Synovial-fluid mononuclear cells were isolated from synovial fluid of SpA patients’ joint punctuations and PBMC were isolated from the blood of the same patient. The cells were cultured for 18 h at 37 °C in wells coated with BT-061 at the indicated concentrations, in untreated wells or they were treated with soluble BT-061 before cultivation. Cells isolated from SF of three individual donors of three independent experiments are shown. From one of these donors also PBMC data are shown. (**B**) Statistical analysis of the percentage of gated T cells that showed CD4 down-modulation as shown in the FACS plots in (**A**). The error bars indicate SEM. (**C**) Either PBMC or synovial fluid cells from the SpA patient #3 were stained with mAbs specific for CD64 (black line) and the respective isotype control (filled grey). (**D**) Whole SF was incubated with 10 μg/ml BT-061 for 18 h at 37 °C and then the CD4 expression was determined cytofluometrically. During the first 4 h whole SF was steadily mixed.

To identify the immune cell subset that conferred CD4-down modulation of BT-061 decorated T cells, co-incubation experiments were performed either with PBMC depleted of selected immune cell subsets, or with the respective MACS-enriched immune cell subsets. Under such conditions, PBMC depleted of B cells conferred a similar CD4 down-modulation as compared with complete PBMC, while isolated B cells did not induce any effects (Fig. 2A, second panel, and B). Interestingly, co-incubation with PBMC depleted of monocytes resulted in significantly reduced CD4 down-modulation, whereas in the reverse experiment purified monocytes induced an even stronger effect than complete PBMC (Fig. 2A, third panel, and B). The high affinity FcγR CD64 expressed by monocytes is a candidate that conferred interaction with BT-061 decorated T cells. Indeed, amongst PBMC only monocytes showed significant CD64 expression (Fig. 2C). On the contrary, monocyte-derived HLA-DR^+^CD14^–^ immature dendritic cells (imDC) did not express CD64 (Fig. 2C) and mediated substantially less CD4 down-modulation than monocytes (Fig. 3D). Since incubation of BT-061 decorated T cells with conditioned supernatant of BT-061 decorated T cells co-incubated with monocytes did not affect CD4 expression (Fig. 2E,F), not soluble factors but merely cell-cell interactions of monocytes with BT-061 decorated T cells presumably via CD64 played the critical role.

Upon washing of CD4 down-modulated T cells and subsequent incubation, CD4 expression recovered (Supplementary Fig. 2C and D). In the presence of the protein biosynthesis inhibitor cycloheximide (CHX) T cells showed reduced CD4 expression and down-modulated CD4 expression did not recover (Supplementary Fig. 2C and D). Thus, homeostatic CD4 expression of T cells as well as recovery of CD4 down-modulation was dependent on *de novo* protein biosynthesis.

### BT-061 treatment of synovial fluid derived cells induces significant CD4 down-modulation of T cells

Patients suffering from bone or joint disturbing autoimmune diseases typically carry auto-reactive T cells at sites of symptomatic inflammation, e.g. the synovial fluid (SF). Therefore, we studied the effect of BT-061 on synovial fluid derived cells and whole synovial fluid obtained from spondyloarthritis (SpA) patients. In an initial experiment BT-061 treatment of SF derived mononuclear cells and of PBMC from the same donors resulted in CD4 down-modulation of T cells, whereas the overall magnitude of the effect detected in SF derived mononuclear cells showed some variations amongst individual donors (Fig. 3A middle and right column and 3B). To determine maximal CD4 down-modulation, SF derived mononuclear cells were incubated in wells coated with BT-061. This experiment revealed a strong and consistent CD4 down-modulation of T cells (Fig. 3A left column and 3B). SF derived mononuclear cells comprised significantly enhanced percentages of CD64^+^ cells when compared with PBMC of the same donor (Fig. 3C). In a final experiment, BT-061 was added to whole SF. In this experiment CD4 down-modulation of T cells was clearly detected (Fig. 3E), which nevertheless was less pronounced than upon incubation of SF derived mononuclear cells in BT-061 coated wells.

### Serum IgG inhibits BT-061 induced CD4 down-modulation of T cells

**Figure 4:**
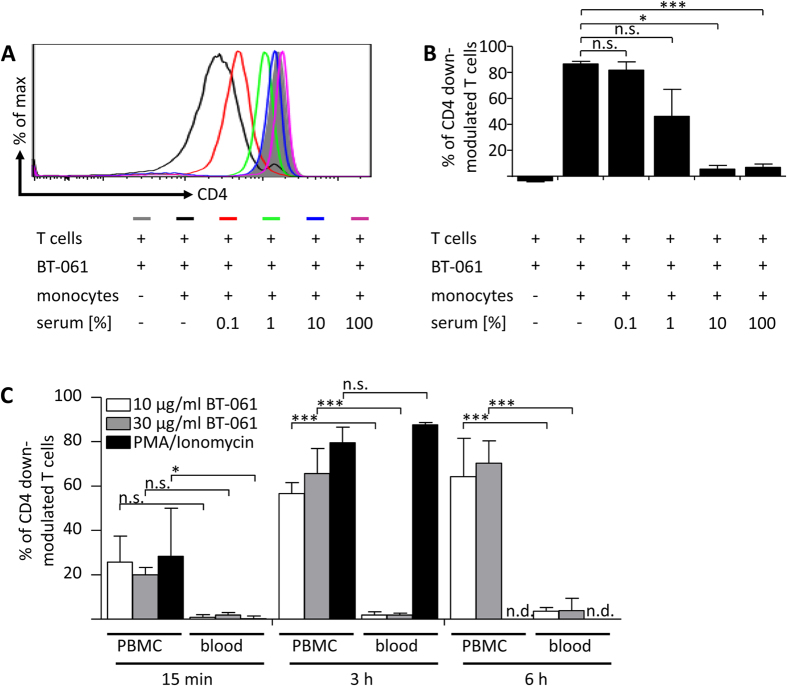
BT-061 addition to T cells co-cultured with serum pre-treated monocytes or to a circulating whole blood system does not induce CD4 down-modulation of T cells. (**A**) Monocytes were incubated for 45 min at 37 °C in medium enriched with the indicated percentages of human serum (0.1% red line, 1% green line, 10% blue line and 100% purple line), or in normal medium (black line). Afterwards the monocytes or medium (filled grey) were added to untreated or BT-061 decorated T cells. After 18  h of incubation at 37 °C, CD4 expression was determined cytofluometrically. Data from one experiment with cells derived from two individual donors are shown. (**B**) Statistical analysis from experiments in (**A**). Error bars indicate SEM. (**C**) Fresh human whole blood was set to rotate in surface-heparinized loops and treated with 1 U/mL heparin and additionally with the indicated concentrations of BT-061 (light and dark grey bars) or PMA (0.05 μg/ml)/Ionomycin (0.75 μg/ml) (black bars). The loops were rotating at a speed of 10 rpm. PBMC were isolated from the same donor and treated with the indicated concentrations of BT-061 (light and dark grey bars) or PMA (0.05 μg/ml)/Ionomycin (0.75 μg/ml) (black bars). Following incubation of whole blood and PBMC at 37 °C for 15 min, after 3 or 6 h samples were harvested and CD4 expression was determined cytofluometrically. Statistical analysis for data of 2 experiments with samples derived from 3 donors. Error bars indicate SEM.

Because in blood CD64 is saturated with monomeric serum IgG^18^, we next addressed whether human serum affected BT-061 induced CD4 down-modulation. Indeed, upon pre-incubation of monocytes with 10% or more human serum BT-061 induced CD4 down-modulation was significantly impaired (Fig. 4A,B). To further address whether BT-061 induced CD4 down-modulation might be induced within the bloodstream, we next employed a human *ex vivo* whole blood loop system^19^. To preserve complement function while prevent clotting, blood from healthy donors was treated with a low dose (1 U/ml) of soluble heparin and was kept in rotation in a Corline heparin inner surface tubing system. In this setting, CD4 down-modulation was not detected after 3 or 6 h of BT-061 incubation (Fig. 4C). Of note, parallel experiments with BT-061 treated PBMC from the same donors showed distinct CD4 down-modulation (Fig. 4C). To confirm functionality of the whole blood system we added PMA/Ionomycin which induced significant CD4 down-modulation (Fig. 4C). To exclude that complement activity played a role, whole blood circulation experiments were also conducted at 10 U/ml of soluble heparin, a concentration which inhibited complement function. Notably, under such conditions overall similar results were obtained as in experiments with 1 U/ml heparin (compare Fig. 4C and Supplementary Fig. 3).

The Fc part of IgG1 mAbs potentially can mediate complement-dependent cytotoxicity (CDC)^20^,^21^. To address whether also BT-061 induced CDC, the absolute number of CD4 expressing cells was determined in the whole blood loop system before and after BT-061 addition. Since at the different time points similar numbers of CD4 expressing T cells were detected (data not shown) we concluded that also under these conditions no CDC was induced.

### Upon BT-061 treatment of tonsil derived mononuclear cells, T cells do not show CD4 down-modulation

**Figure 5:**
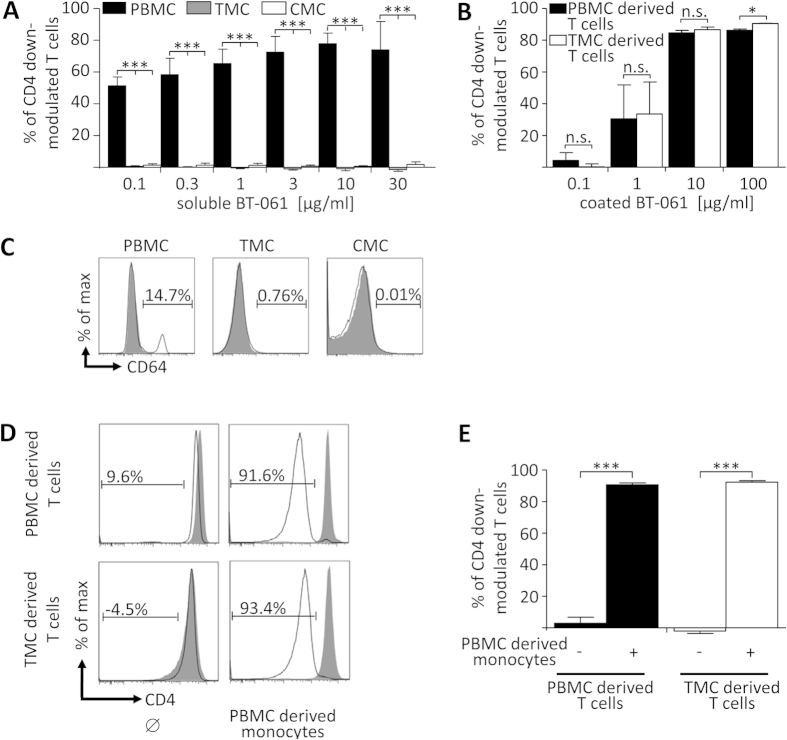
BT-061 treatment of tonsil derived mononuclear cells does not induce CD4 down-modulation of T cells. (**A**) PBMC (black bars), TMC (grey bars) or CMC (white bar) were treated with the indicated concentrations of BT-061 for 45 min at 4 °C. Upon washing, the cells were cultivated for 18 h at 37 °C and then CD4 expression was determined cytofluometrically. Statistical analysis of 2 experiments with cells from 3–5 donors (PBMC), 4 experiments with cells from 6 donors (TMC) and 3 experiments with cells from 5 donors (CMC). (**B**) T cells isolated from PBMC or TMC derived from the same donor were cultured for 18 h at 37 °C in wells coated with BT-061 at the indicated concentrations or in untreated wells. Statistical analysis of 2 experiments with cells from 3 different donors. (**C**) PBMC, TMC or CMC were stained for CD64 (filled grey) and isotype control (black line). The shown histograms for PBMC are representative for data obtained by 3 experiments with cells from 3 donors and with TMC for 3 experiments with cells from 4 donors. (**D**) T cells isolated either from PBMC or TMC from the same donor each were treated with BT-061 or medium. Upon washing, the cells were cultured for 18 h at 37 °C in medium or together with syngeneic monocytes isolated from PBMC. Then CD4 expression was determined cytofluometrically. Representative data from 3 experiments with cell from 3 different donors are shown. (**E**) Statistical analysis from all experiments as shown in (**D**). Error bars indicate SEM.

Since BT-061-decorated T cells could not interact with monocytes in the blood due to the saturation of CD64 by serum IgG, we next sought to investigate the impact of BT-061 treatment on cells derived from secondary lymphoid organs. To this end, single cell suspensions from human tonsils (TMC) and tonsil-derived capsular mononuclear cells (CMC) were treated with soluble BT-061. Of note, BT-061 concentrations that in PBMC induced massive CD4 down-modulation did not mediate significant effects on T cells in TMC or CMC (Fig. 5A). Nevertheless, upon incubation of TMC derived T cells in wells coated with BT-061 a similar CD4 down-modulation was observed as with PBMC derived T cells (Fig. 5B). This indicated that absence of BT-061 induced CD4 down-modulation in TMC and CMC was not associated with a certain stimulation status of the T cells. Instead, in TMC and CMC no CD64 expressing cells were present that could interact with BT-061 decorated T cells (Fig. 5C and^22^). Interestingly, TMC derived T cells incubated with syngeneic PBMC-derived monocytes showed restored CD4 down-modulation (Fig. 5D,E). These data demonstrated that BT-061 treated TMC and CMC did not show CD4 down-modulation because these cell preparations comprised only very few, if any, monocytes or other CD64 expressing cells.

## Discussion

Here we addressed the role of Fcγ receptor (FcγR) interactions in BT-061 induced CD4 down-modulation of T cells. We discovered that BT-061 binding to purified T cells alone did not confer CD4 down-modulation and that the additional interaction of BT-061 decorated T cells with CD64 expressing cells was needed in order to induce robust CD4 down-modulation. Correspondingly, BT-061 treated PBMC comprising approximately 6.15% monocytes, which amongst PBMC were the only abundant CD64^+^ immune cell subset, showed significant CD4 down-modulation, whereas PBMC depleted of monocytes did not show this effect. Notably, in a circulating whole blood system BT-061 mediated CD4 down-modulation was not detectable, presumably due to CD64 saturation with serum derived IgG. Interestingly, tonsil derived mononuclear cells obtained from the centre (TMC) or the capsul (CMC), were devoid of CD64^+^ immune cells and upon BT-061 treatment both cell preparations did not show CD4 down-modulation of T cells. In contrast, BT-061 treatment of synovial fluid (SF) derived immune cells as well as of whole SF, both preparations containing high percentages of CD64^+^ cell, showed strong CD4 down-modulation of T cells.

Since BT-061 is a monomeric IgG1 molecule with bivalent specificity^23^, it is expected that upon binding of CD4 on the T cell surface cross-linking of two receptors occurs. Nevertheless, our data demonstrated that bivalent CD4 binding was not sufficient to induce CD4 down-modulation. Instead, an additional interaction with Fcγ receptor expressing cells was needed. This can be explained by the fact that CD4 down-modulation was dependent on agonistic signaling that was boosted by FcγR interaction as previously observed for human T cells treated with a superagonistic anti-CD28 mAb^8^.

Experiments with PBMC depleted of selected cell subsets or with purified single cell subsets revealed that CD64^+^ monocytes were necessary and sufficient to confer massive CD4 down-modulation of BT-061 decorated T cells. Of note, unlike conditions detected for TGN1412^8^, B cells did not confer CD4 down-modulation. Using monocytes-derived imDC that are CD32B^+^, only minor CD4 down-modulation was detected, indicating only limited effects of CD32B. Thus, the interaction with the high affinity FcγR CD64 expressed by monocytes played a central role in the induction of CD4 down-modulation of BT-061 decorated T cells. Our experiments with the inhibitor PP1 revealed partial Lck dependence of CD4 down-modulation. This indicated that CD4 down-modulation of T cells in BT-061 treated PBMC was triggered by agonistic signaling. Interestingly, even application of high concentrations of PP1 did not entirely inhibit BT-061 induced CD4 down-modulation, although already low PPI concentrations quantitatively block Lck activation^24^. Furthermore, it was demonstrated that in addition to the activation pathway through TCR, Lck also contributes to the down-regulation of T cell activation and the induction of cytokine production^25^. Thus, in BT-061 mediated signaling Lck played an important but not exclusive role. Interestingly, similar to constitutive CD4 expression also CD4 recovery of down-modulated T cells was dependent on protein biosynthesis. Thus CD4 recycling from internal pools did not contribute to CD4 recovery.

The above notion that CD4 down-modulation of BT-061 cells was dependent on cell-cell interaction with CD64^+^ cells was further supported by experiments with mononuclear cell preparations of tonsils that were devoid of CD64^+^ cells and did that not show BT-061 mediated CD4 down-modulation. Importantly, T cells isolated from tonsil derived cell preparations showed strong CD4 down-modulation when incubated in BT-061 coated wells or upon BT-061 treatment and co-incubation with blood derived syngeneic monocytes. Thus, absence of CD4 down-modulation in BT-061 treated preparations of tonsil-derived mononuclear cells was not associated with an enhanced pre-activation status of the T cells that differed from that of blood derived T cells^8^,^26^. Instead absence of CD4 down-modulation was simply due to the absence of the cross-linking cellular partner.

CD64^+^ monocytes most efficiently mediated CD4 down-modulation of BT-061 decorated T cells, and considering the observation that in presence of high concentrations of serum IgG BT-061 mediated CD4 down-modulation was significantly reduced, it is questionable whether in the blood stream monocytes can still interact via CD64 with IgG decorated cells. Previously Laurent *et al.* published that monocytes from RA patients showed moderately elevated percentage of CD64^+^ cells, while the MFI of CD64 expressed on that particular cell subset was increased compared with healthy controls^27^. In contrast, two other studies did not detect CD64 upregulation of monocytes in the context of autoimmune diseases^28^,^29^. Additionally, it has been described that other immune cell subsets such as NK cells showed massive CD64 upregulation under proinflammatory conditions, e.g. interferon (IFN)-γ treatment^30^. Of note, since intensive cell-cell interaction of CD64^+^ cells with BT-061 decorated T cells is a prerequisite to induce CD4 down-modulation, the blood stream presumably is not the compartment where CD4 down-modulation is primarily induced. This hypothesis was further supported by the observation that CD4 was not down-modulated upon BT-061 injection into a circulating whole blood system. In this system, the addition of low dose heparin (1 U/ml) ensures that complement is active while clotting is prevented, whereas high dose heparin (10 U/ml) also inhibits the function of the complement system. Studies in the presence of 1 and 10 U/ml heparin revealed that the absence of BT-061 mediated CD4 down-modulation in the circulating whole blood system was not due to interactions of the complement system with BT-061, which is an antibody of the IgG1 class that potentially has the capacity to interact with the complement system. CD4^+^ T cell counts did not drop in the system containing active complement proteins indicating that BT-061 does not confer major complement-dependent cytotoxicity (CDC), which is in line with previous data^14^. Despite BT-061 is expressed in an IgG1 format, also ADCC did not play a role because in the CD4 down-modulation assay total counts of CD3^+^ cells stayed constant.

The experiments with SF of SpA patients demonstrated that within inflamed compartments, where auto-reactive T cells and increased percentages of CD64 expressing cells are present, BT-061 mediated CD4 down-modulation is clearly detectable. Therefore it is likely that upon BT-061 treatment of patients with autoimmune diseases CD4 down-modulation of T cells is not induced systemically within the blood stream or in secondary lymphoid organs, but instead it is primarily induced locally at inflamed sites, e.g. within SF, to which auto-reactive T cells as well as different CD64^+^ inflammatory immune cells subsets are recruited. Although in such compartments enhanced IgG concentration are found in RA patients^31^, cells seem to express even further enhanced CD64 levels^32^. Furthermore, it was shown in SF that neutrophils as well as synovial mast cells can show upregulated CD64 expression in case of inflammation^33^,^34^,^35^. Thus, considering the reduced circulation of the synovium, it is conceivable that in spite of the presence of enhanced IgG levels, which nevertheless do not exceed serum levels^31^,^36^, BT-061 induced CD4 down-modulation of BT-061 decorated T cells takes place at inflammatory sites. This notion is further supported by the observation that at inflammatory sites CD64 expression is enhanced compared with recirculating blood monocytes of the same donors. A similar site-specific action of mAb that interact with FcγR on monocytic cells was recently discovered also in the cancer field. In mouse experiments interaction of FcγRIV with CD11b^+^ macrophages that were highly enriched within tumor tissue compared with secondary lymphoid tissues such as lymph nodes conferred ADCC of infiltrating T_reg_ cells^37^,^38^.

Nevertheless, in a more recently performed clinical trial CD4 down-modulation was detected also on blood derived T cells of BT-061 treated patients (data not shown). It is possible that in these individuals CD4 down-modulation took place within inflamed sites from where the T cells then re-shuttled into the blood stream. It will be a matter of future experiments to verify this hypothesis experimentally, e.g. by comparing CD4 down-modulation of T cells in BT-061 treated patients derived either form inflammatory sites or from blood. Under such conditions it would be particularly attractive to study T cells subsets such as T_regs_ that were reported to be mainly triggered by BT-061 in *in vitro* studies^16^.

The data presented here are compatible with the hypothesis that upon BT-061 treatment basically all CD4^+^ T cells are decorated, whereas in contrast to T cells within secondary lymphoid organs or within the blood stream, primarily T cells recruited to local inflammatory sites can interact with CD64^+^ cells and then show CD4 down-modulation. In contrast, the conditions within SF allow the induction of CD4 down-modulation. Although in RA patients in this compartment higher amounts of IgG are present, immune cell subsets upregulate CD64, e.g. such as HLA-DR^+^ CD14^+^ macrophages, which further facilitates cell-cell interactions. Additionally, recirculation of the synovial fluid is reduced when compared with blood. This is not only due to the fact that the synovial fluid is a separated compartment but also that in particular in patients with rheumatic disorders the synovial fluid shows a very high viscosity. Thus, our results underscore the need not only to analyze interactions of a given mAb with single FcγR on the protein level, but also to address whether the immunological microenvironment is appropriate to support FcγR mediated cell-cell interactions.

## Methods

### Reagents and antibodies

LIVE/DEAD® Fixable Aqua Dead Cell Stain Kit for 405 nm excitation (Invitrogen). AccuCheck Counting Beads (Molecular probes). Cycloheximide (CHX) (Sigma-Aldrich). Phosphoprotein phosphatase (PP1) (Biaffin GmbH). Fluochrome labelled antibodies CD4-FITC (clone SK3), CD8a-PE.Cy7, and HLA-DR-APC.Cy7 (BioLegend), CD3-APC, CD4-PE (clone SK3), CD19-PE, CD14-V450, CD56-FITC, CD64-FITC and Iso IgG1κ-FITC (BD) and CD56-APC (ImmunoTools).

### Isolation and differentiation of human immune cells

Peripheral blood mononuclear cells (PBMC) from buffy coats of healthy donors (Blood Donor Center, Springe, Germany) and cells from synovial fluid of SpA patients’ joint punctuations (in- and outpatients of the Clinic for Immunology and Rheumatology, Hannover Medical School, after informed written consent; the study was approved by the local ethical committee (ethical approval number 5582) and the experiments were performed according to the approved guidelines) were isolated by Ficoll (Biochrom) density gradient centrifugation. The human tonsil specimens incurred in context of surgical interventions (ethical approval number 1916–2013, the experiments were performed according to the approved guidelines). To prepare tonsillar mononuclear cells (TMC) the gentle MACS (Miltenyi Biotech) was used. For tonsil-derived capsular mononuclear cells (CMC) the tonsil enclosing tissue was digested by using Liberase TL (ROCHE). For cultivation of TMC and CMC X-VIVO 15 medium (Lonza) enriched with penicillin (f.c. 100 U/ml) and streptomycin (f.c. 100 μg/ml) was used. T cells, B cells, and monocytes were purified using the Pan T cell Isolation kit II, CD19 MicroBeads, or CD14 Microbeads (Miltenyi Biotech), respectively. For cell separation AutoMACS Pro (Miltenyi Biotech) was used. Monocyte derived immature dendritic cells (imDC) were differentiated as described before^39^.

### Whole blood and plasma experiments

Plastic tubings made of polyvinylchloride (inner diameter 4 mm) with a heparin surface (Corline Systems) were applied as previously described^19^. Briefly, blood was collected into a surface-heparinized Falcon tube containing heparin (Leo Pharma) at a final concentration of 1 (low dose) or 10 (high dose) U/ml heparin. The loop was filled with 2 ml of blood and subsequently reagents of choice were added. Surface-heparinized tubes were formed into loops using a surface-heparinized connector (loop length 200 mm). Loops were set to rotate at 10 rpm in a 37 °C chamber. At 15 min, 3 or 6 h loops were taken out of the chamber, samples were collected, and loops were closed and set to rotate again. Blood samples were immediately transferred into tubes containing EDTA (10 mM final concentration) and stored on ice. Platelets were counted with a Coulter AcT diff Hematology Analyser (Beckman Coulter).

### Flow cytometric analysis

FACS data were acquired on a LSRII FACS analyser (BD) and processed using FlowJo software (Tree Star Inc.). To avoid unspecific binding, 1% of Gamunex (Talecris) was added and cells were fixed in 1% paraformaldehyde (PFA) (Merck). For immune cell subsets analysis 2 × 10^5^ cells were stained for the lineage specific markers.

### Immunological assays

Cell preparations were incubated at 4 °C with BT-061 at the indicated concentrations for 45 min in X-VIVO 15 at 1 × 10^6^/ml. Then, cells were washed and T cells were seeded at 1 × 10^6^/ml, whereas PBMC, TMC or cells isolated from SF were seeded at 4 × 10^6^/ml. For co-cultures of T cells with syngeneic immune cells, the latter ones were added at 4 × 10^5^/ml. T cells were incubated in wells coated with BT-061 at 1 × 10^6^/ml. For mAb stimulation of SF, BT-061 was added at a final concentration of 10 μg/ml. SF samples show an enhanced viscosity and therefore had to be permanently mixed to ensure mAb distribution.

To test for factors secreted by monocytes co-cultured with BT-061 decorated T cells, 100 μl of the supernatant was harvested and added to 2 × 10^5^ T cells of the same donor. Then T cells were seeded at 1 × 10^6^/ml. To pretreat monocytes with serum, heat-inactivated human AB serum was used (c.c. pro). Monocytes were seeded at 8 × 10^6^/ml in X-VIVO 15 and serum was added at indicated concentration. After 45 min at 37 °C, 2 × 10^5^ T cells were added at 2 × 10^6^/ml. To coat the antibody, the reagent was diluted in coating buffer (0.1 M Na_2_HPO_4_ × 2H_2_O (Merck), pH 9.0) at indicated concentrations.

### Statistics

Statistical analysis was conducted using one-tailed student t test for Figs 1F, 2D/F and 5B/E or One-way-ANOVA with correction for multiple comparisons for Figs 1D, 2B, 4B and C, and 5A. P-values of <0.05 were considered statistically significant (<0.05=*; <0.01=**; <0.001 = ***). Data are presented as mean with ± SEM, whereas bias correction for the SEM of the normal distribution was conducted when sample number was below 10. GraphPad Prism (Version 5.02 and 6; GraphPad) was used for all statistical analyses. To determine the percentage of gated T cells that showed CD4 down-modulation a program was established that defines a standardized gate comprising 1% of events of the stained peak. The program automatically subtracts a varying CD4 negative background contributed by CD3^+^CD8^–^CD4^–^ cells.

## Additional Information

**How to cite this article**: Vogel, S. *et al.* Antibody induced CD4 down-modulation of T cells is site-specifically mediated by CD64^+^ cells. *Sci. Rep.* **5**, 18308; doi: 10.1038/srep18308 (2015).

## Supplementary Material

Supplementary Data
