# Supplementary material for: Antibody induced CD4 down-modulation of T cells is site-specifically mediated by CD64+ cells
Source: Sci Rep. 2015 Dec 16;5:18308. doi: 10.1038/srep18308 (PMC4680940; doi:10.1038/srep18308)
Supplement: Supplementary Data [file srep18308-s1.pdf]

Antibody induced CD4 down-modulation of T cells is site-specifically mediated by CD64⁺ cells

Stephanie Vogel, Elena Grabski, Daniela Buschjäger, Frank Klawonn, Marius Döring, Junxi Wang, Erika Fletcher, Ingo Bechmann, Torsten Witte, Martin Durisin, Burkhart Schraven, Sara M. Mangsbo, Kurt Schönfeld, Niklas Czeloth, Ulrich Kalinke

Supplementary Figure 1

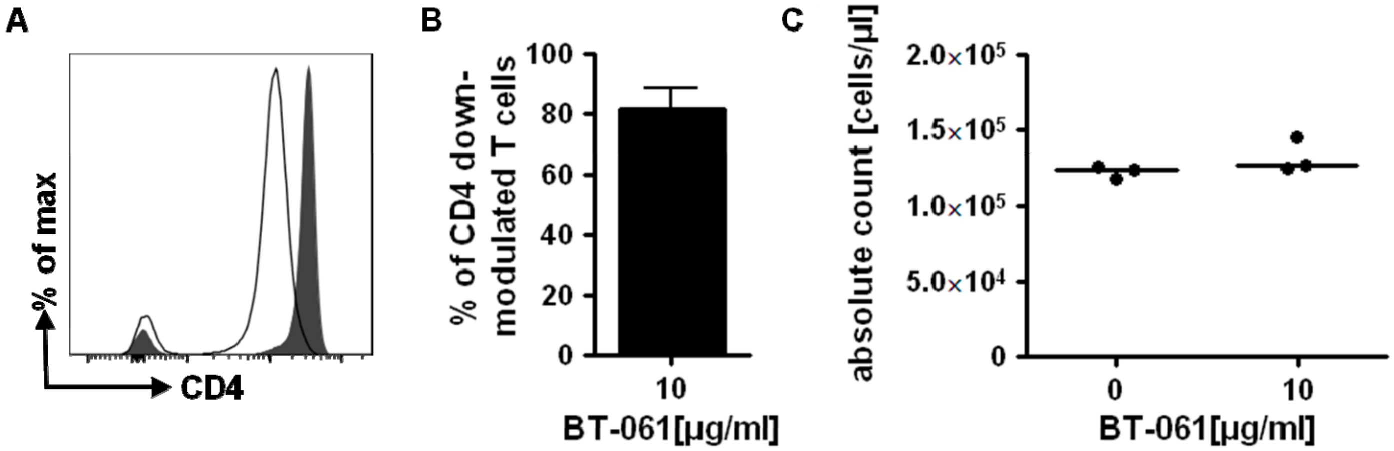

Supplementary Figure 1: BT-061 treatment of PBMC induces CD4 down-modulation and no depletion of T cells. (A) PBMC were treated with 10 µg/ml of BT-061 for 45 min at 4°C and were then cultivated for 18 h at 37°C. After this incubation CD4 expression of T cells was determined cytofluometrically. One donor out of three is shown. (B) Statistical analysis from all donors. (C) PBMC were treated with BT-061 (10 µg/ml) for 45 min at 4°C or left untreated and then cultivated for 18 h at 37°C. Absolute numbers of $CD3^+$ cells was determined cytofluometrically using counting beads.

Supplementary Figure 2

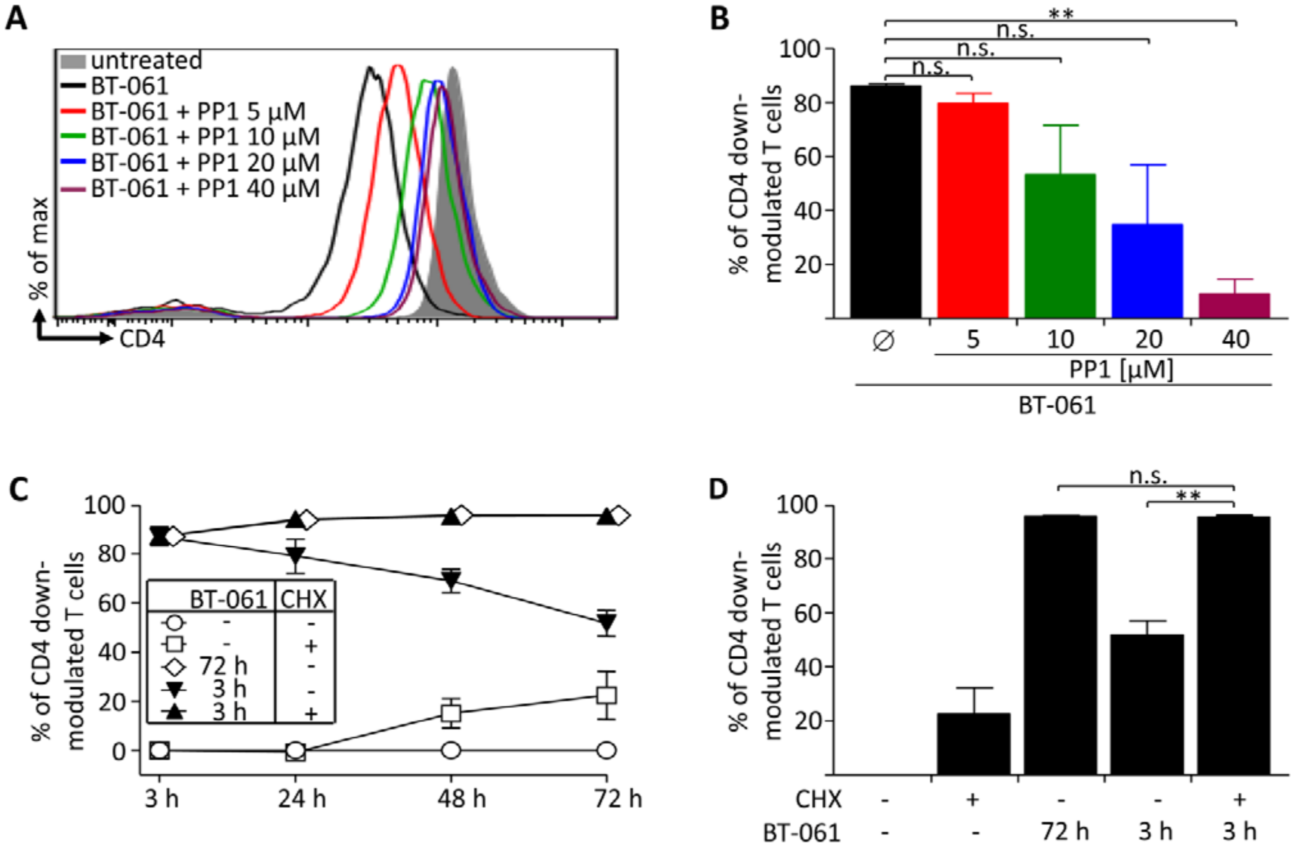

Supplementary Figure 2: CD4 down-modulation of T cells induced by immobilized BT-061 is dependent on Lck activation, whereas recovery of CD4 expression is dependent on *de novo* protein biosynthesis. (A) PBMC were treated for 45 min at 37°C with PP1 at the indicated concentrations. Cells were cultivated for 18 h at 37°C in medium, medium supplemented with BT-061 (10 µg/ml), or medium supplemented with PP1 (at the indicated concentrations), then CD4 expression was determined cytofluometrically. One experiment with cell derived from 3 donors is shown. (B) Statistical analysis from all experiments as shown in (A). (C) MACS enriched CD4$^+$ T cells were incubated for 3 h in untreated wells, in medium supplemented with CHX (10 µg/ml) or in wells coated with BT-061 (10 µg/ml). Untreated cells were further cultivated in untreated wells (white circle), whereas CHX pre-treated cells were cultivated in medium supplemented with CHX (10 µg/ml) (white square), and BT-061 treated T cells were cultured on immobilized BT-061 (10 µg/ml) (white diamond), medium (black triangle pointing down) or medium supplemented with CHX (10 µg/ml) (black triangle pointing up). In experiments with CHX, every 24 h CHX was added at a concentration of 10 µg/ml. CD4 expression was determined after the indicated time cytofluometrically. Statistical analysis of one experiment with cells derived from 3 different donors. (D) Depiction of values obtained after 72 h incubation based on the data also shown in (B). Error bars indicate SEM.

Supplementary Figure 3

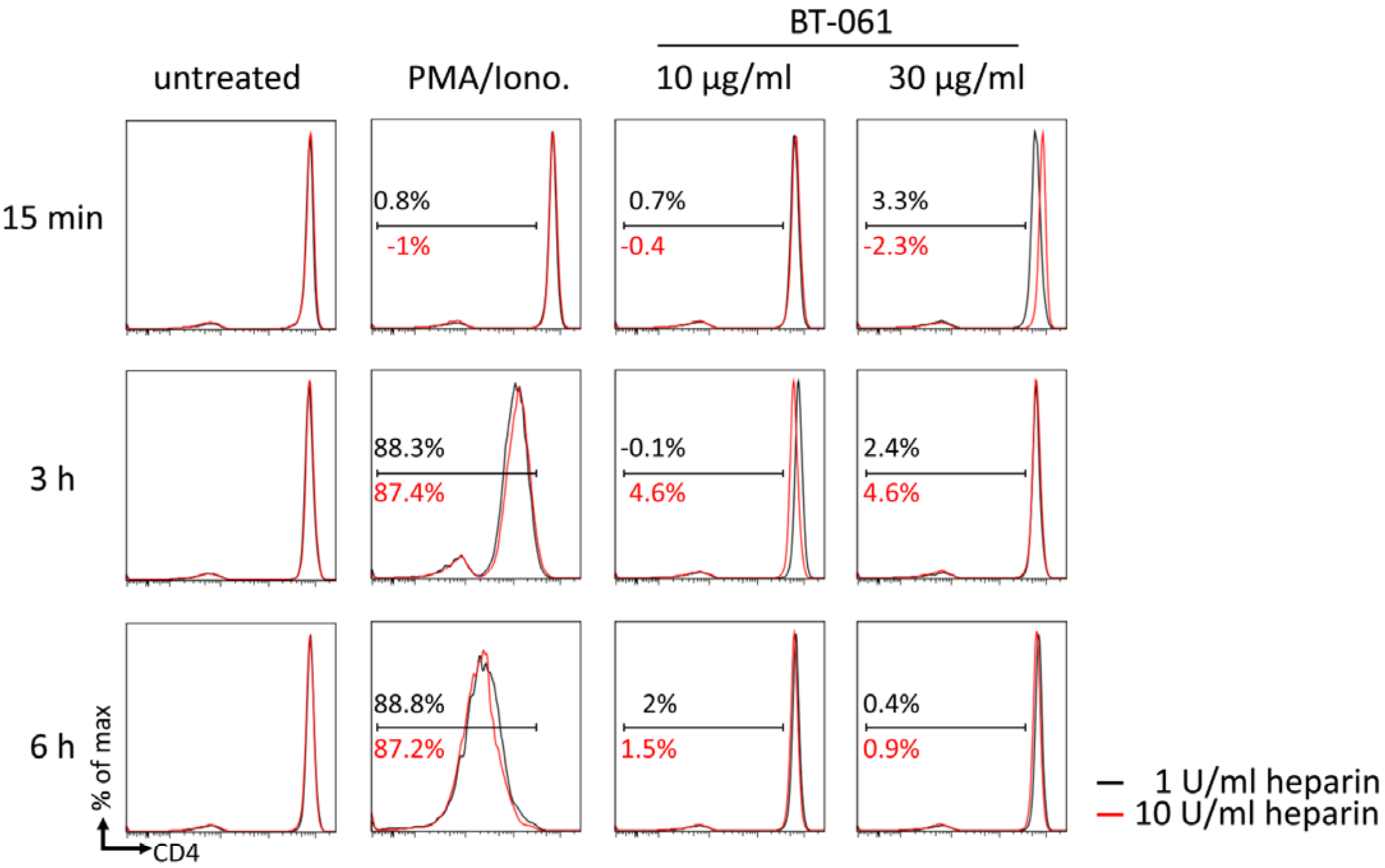

Supplementary Figure 3: BT-061 mediated CD4 down-modulation in a circulating whole blood system is not abrogated due to interaction with the complement system. Fresh human whole blood was set to rotate in surface-heparinized loops made of polyvinylchloride and treated with 1U/ml (black line) and 10 U/ml (red line) heparin and additionally with the indicated concentrations of BT-061 or PMA (0.05 µg/ml)/Ionomycin (0.75 µg/ml). The loops were placed on a wheel rotating at a speed of 10 rpm. Following an incubation of whole blood at 37°C for 15 min, 3 or 6 h samples were harvested and then CD4 expression was determined cytofluometrically.
